# The historical transformation of the classification of personality disorders

**DOI:** 10.1192/j.eurpsy.2023.365

**Published:** 2023-07-19

**Authors:** N. Schierenbeck

**Affiliations:** IZWT-Interdisciplinary Centre for Science and Technology Studies, Bergische Universität Wuppertal, Wuppertal, Germany

## Abstract

**Introduction:**

The classification system of personality disorders underlies historical transformation processes. In 1980 the DSM-III introduced a categorial approach to systematize personality disorders. With further research the alternative DSM-5 Model for Personality Disorders with categorial and dimensional elements was introduced in 2013. Latest changes manifest with a dimensional classification system of personality disorders in ICD-11. The transition from a categorial towards a dimensional classification system of personality disorders is framed as a ‘paradigm shift’.

**Objectives:**

Aim of this investigation is to describe these transitions and analyze if these changes correspond to the term ‘paradigm’ in Thomas Kuhn sense (Kuhn Uni. of Chicago Press 1962), or are more a form of continuous and gradual change. Particularly I examine how knowledge transforms within the disciplines of psychiatry and psychology, and how the arena in which the scientific discussions are negotiated is constituted.

**Methods:**

To analyze the historical transformation processes within the scientific communities a qualitative reconstructive approach is chosen. An extensive bibliometric literature research in the databases Web of Science, PubMed and JSTOR is conducted. Back and forward citation are taken into account until a dense set of primary and secondary sources from 1970-2022 is accumulated.

**Results:**

Results show that the transition from a categorial towards a dimensional classification system of personality disorders is not a classical ‘paradigm shift’ in Thomas Kuhn sense. The changes have the character of gradual and fluid transitions. New knowledge is added to older knowledge and established insights are not replaced fully. Researchers from the disciplines of psychiatry and psychology argue for a dimensional approach and show scientific evidence, which makes them to driving forces behind the transformation process. Even though in the scientific communities are contrary voices who argue for a moderate approach and emphasize the practical applicability of diagnostic manuals.

**Conclusions:**

In summary scientific knowledge in the disciplines of psychiatry and psychology changed. Researchers from the different scientific communities had an impact on political decision-making processes. To understand the driving forces fully and how scientific knowledge interacts with politics, the institutions APA (American Psychiatric Association) and WHO (World Health Organization) and their institutional procedures of the revision process must be investigated in more detail.

**Disclosure of Interest:**

None Declared

